# Protocol for Low-level laser therapy in traumatic ulcer after troncular anesthesia: Case report in pediatric dentistry

**DOI:** 10.4317/jced.56176

**Published:** 2020-02-01

**Authors:** Thais-Apolinário Calazans, Priscila-Hernandez de Campos, Ana-Valesca-Gurjão Melo, Alba-Valeska-Alves Oliveira, Stella-Ferreira Amaral, Michele-Baffi Diniz, Tatiane-Fernandes Novaes

**Affiliations:** 1 Post-graduate Program in Dentistry, Cruzeiro do Sul University; 2 Pediatric Dentistry,Center of studies and researches in Dentistry /COESP

## Abstract

The aim of this study was to report a protocol of use for low-level laser therapy (LLLT) in traumatic ulcer in the lower lip after inferior alveolar nerve block anesthesia (IANBA). A 3-year-old patient, male, undergoing treatment of carious lesions was submitted to an indirect pulp capping in tooth 74 under IANBA. The procedure was completed without intercurrences, but on next day, the child presented extensive traumatic ulcer in the left lower lip, with complaint of pain. Two sequential applications with LLLT were applied in punctual mode under pressure around the lesion. After 1 week, the mother reported significant improvement. After 30 days, the lesion was fully healed. In conclusion, LLLT promoted rapid analgesia and healing, being a good treatment alternative for traumatic ulcer after troncular anesthesia.

** Key words:**Local anesthesia, soft tissue injuries, laser therapy, children.

## Introduction

The use of local anesthesia in dentistry has the advantage of keeping the patient conscious during treatment while inducing temporary loss of sensation, including pain, in a part of the body (1,2). Regardless of the type of local anesthetic, the postoperative effect, especially in the soft tissues, may last for several hours (2). Hence, traumatic lesions may occur in these tissues after inducing local anesthesia, especially after inferior alveolar nerve block (3,4). Pediatric patients are commonly affected because they do not understand the effects of local anesthesia and tend to suck and bite the affected area because of curiosity associated with numbness, or unintentionally during eating or sleeping (2,5,6), possibly resulting in laceration and swelling of the tissues (1).

Studies reported that this type of traumatic injury to the lips, cheeks, and tongue occur after inducing local anesthesia in 4-17.8% of the patients, commonly between 0 and 12 years of age, with a higher prevalence in pre-school children (1,6,7). Most lip and cheek injuries of this nature are self-limiting and heal without complications, although bleeding and infection may occur. In addition, major discomfort affects the daily life of the affected patients (2,8). For the management and treatment of these traumatic injuries, oral antiseptic solutions and medications such as anti-inflammatory drugs and systemic analgesics can be used (9-11). An useful non-drug alternative would be LLLT to aid tissue healing and analgesia (12).

Low-intensity infrared laser is considered most effective for analgesia because it acts by increasing the metabolism, proliferation, maturation, and amount of granulation tissue and decreasing the inflammatory mediators, thereby initiating the healing process (13). However, the use of LLLT for the treatment of traumatic ulcers after local anesthesia in pediatric dentistry has not yet been explored in the literature. Thus, this study aimed to present a protocol of use for LLLT for the treatment of traumatic ulcer in the lower lip after inferior alveolar nerve block anesthesia (IANBA) in a 3-year-old child.

## Case Report

A 3-year-old boy visited the Pediatric dental clinic of COESP Faculty (Center of Studies and Researches in Dentistry), João Pessoa-PB, Brazil, accompanied by his mother, with the complaint of dental caries. An informed consent form was signed before the treatment.

Clinically, the child had extensive carious lesions on the deciduous teeth but with no pulp involvement. The proposed treatment plan included glass ionomer cement restorations, topical fluoride application, diet and oral hygiene maintenance. Indirect pulp capping (IPC) of tooth 74 was performed under inferior alveolar nerve block anesthesia (IANBA) using 2% lidocaine with 1:100,000 epinephrine and isolation of the operative field. At the end, the mother and child were instructed about post-anesthetic care, such as restriction on biting or sucking the lip/cheek, avoiding the ingestion of hot substances.

**Figure 1:**
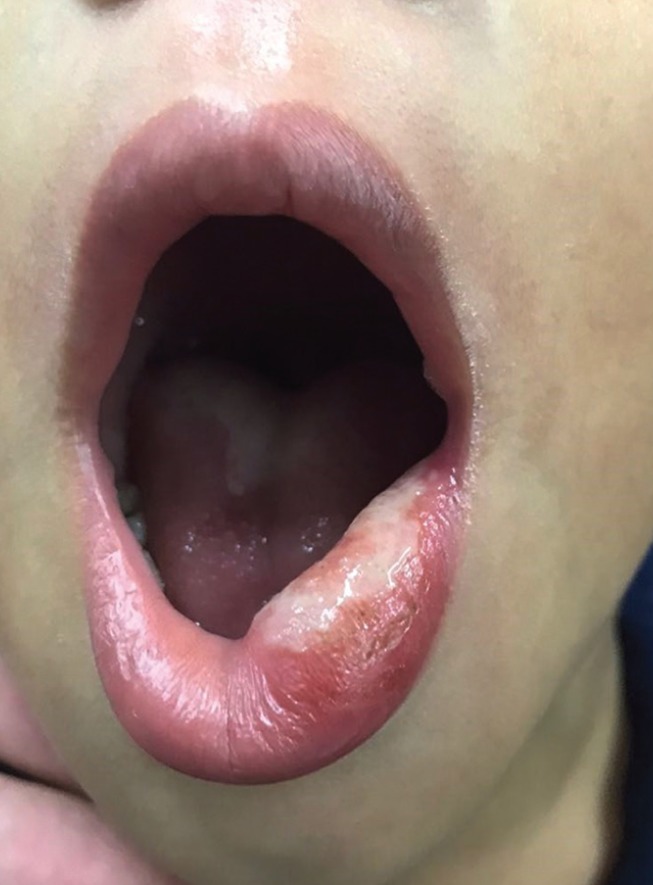
Clinical appearance of traumatic ulcer in the lower lip.

The next morning, the child returned to the dental clinic, complaining of pain, difficulty in eating, and a lip ulcer. Clinical examination revealed a large traumatic ulcer on the left lower lip, with a whitish plaque (Fig. 1). The mother reported that she was unable to prevent the child from biting his lip after the procedure when he fell asleep on the way home.

**Figure 2:**
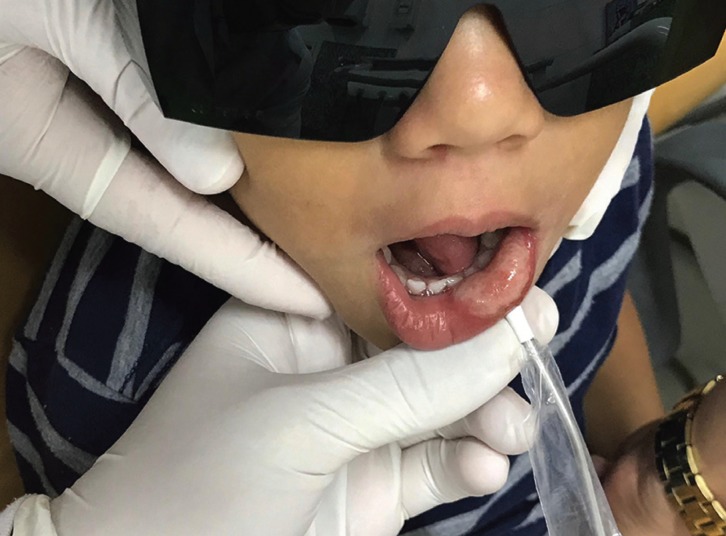
Laser therapy done with a ponctual application under pressure around the ulceration.

**Figure 3:**
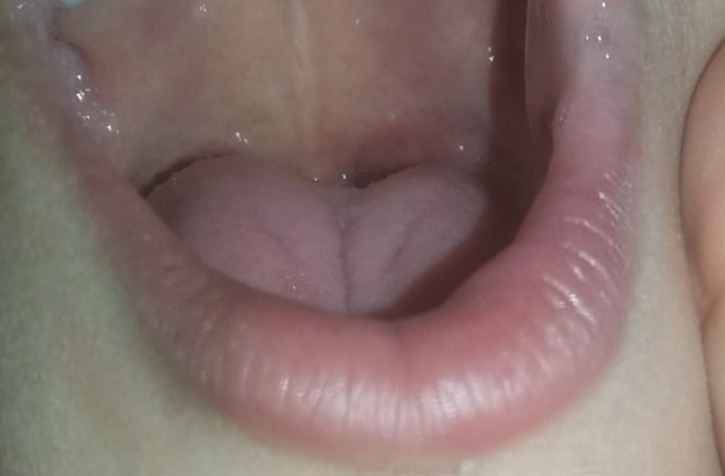
Clinical appearance of the lower lip after 30 days.

The proposed treatment was the application of LLLT for analgesia, inflammatory process control, and tissue repair. The Protocol of use of LLLT in 

our School is: Two sequential applications of infrared diode laser (Whitening Lase II, DMC Equipamentos Ltda., São Carlos, Brazil) performed in the continuous mode, at a wavelength of 808 nm, 100 mW power, and energy density of 105 J/cm2 for 5 seconds precisely around the entire lesion extension, with the fiber perpendicular to the tissue under pressure (Fig. 2).14 As a supplement to the treatment, was prescribed 0.12% chlorhexidine gluconate for the hygiene of the ulcerated area. After 1 week, over the phone, the patient’s caregiver reported significant improvement in the condition, and pain medication was not needed because the child was able to eat normally. After 30 days, the child attended the maintenance appointment and showed no signs of lower lip ulceration (Fig. 3). The patient continued the usual dental treatment planned.

## Discussion

After dental treatment of IPC performed under local anesthesia, the child may bite the lower lip out of curiosity associated with numbness, or unintentionally during eating and sleeping (1). Chi *et al.* (5) observed that 13% children suffered soft tissue trauma following unilateral or bilateral mandibular local anesthesia. Accidental lip biting may occur during the postoperative period while eating or sleeping, as presented in this case report, wherein the child fell asleep after dental treatment under local anesthesia by inferior alveolar nerve block, keeping the adult from preventing the child’s self-injury, although the parent had been informed about the need to monitor the child’s behavior to avoid biting or sucking the lip, cheeks, and tongue. Illustrations of example cases shown to the persons responsible for the child may emphasize the importance of this observation during the numbness period (2).

Most traumatic soft tissue lesions are self-limiting and heal without complications but involve massive discomfort, interfering with the day-to-day activities such as eating (2,8). The patient in this case report complained of pain and inability to ingest any liquid or solid food. Thus, pain prevention and control during and after the dental procedure is essential to nurture the patient’s relationship with the dental surgeon, create confidence, alleviate fear and anxiety, and promote a positive attitude (2). Consequently, in this case, immediate laser therapy was indicated for both analgesia and tissue repair.

Low-intensity laser therapy aims at relieving painful symptoms because of its painkiller properties (12). The therapeutic laser has no direct curative effect but favors tissue repair in the injured region through cellular biostimulation (13). In this case, the mother reported rapid ulcer healing along with decreased pain.

Although low-power laser therapy has good results, most dentists do not own the device, mainly because of the high acquisition and maintenance costs. In this case report, the treatment was performed in the School Clinic in a Faculty that already had the device, therefore, we selected this treatment modality. An alternative, as reported in the work of Arenas and Rivera (10), would be drug therapy with painkillers, such as ibuprofen in oral suspension, 200 mg/5 mL, at dosages of 10 mg/kg every 8 hours for 5 days and clinical control every two days, with full trauma resolution at 10 days after medication and orientation.

According to Lizarelli (14), the protocol recommended for analgesia and reduction of inflammation are two sessions of laser therapy in an interval of 24 hours. In the case presented, due to the monthly periodicity of the course, both applications were carried out consecutively in the same session. In addition, we performed spot irradiation in contact with the tissue under pressure, which is the most recommended method because it presents less variability (12).

In pediatric dentistry, self-injury presents frequently, especially after nerve block. So, in the case presented, low-intensity laser therapy promoted rapid analgesia, healing, and anti-inflammatory effects. Thus, it is a good treatment alternative for traumatic ulcers after truncal anesthesia.
